# If you can't comply with dialysis, how do you expect me to trust you with transplantation? Australian nephrologists' views on indigenous Australians' 'non-compliance' and their suitability for kidney transplantation

**DOI:** 10.1186/1475-9276-11-21

**Published:** 2012-04-18

**Authors:** Kate Anderson, Jeannie Devitt, Joan Cunningham, Cilla Preece, Meg Jardine, Alan Cass

**Affiliations:** 1 The George Institute for Global Health, Sydney, Australia; 2 Menzies School of Health Research, Charles Darwin University Darwin, Australia, Sydney Medical School, University of Sydney, Sydney, Australia; 3 The George Institute for Global Health, Sydney, Australia, Sydney Medical School, University of Sydney, Sydney, Australia

**Keywords:** Kidney transplantation, Indigenous peoples, Aboriginal and Torres Strait Islander, Compliance

## Abstract

**Introduction:**

Indigenous Australians suffer markedly higher rates of end-stage kidney disease (ESKD) but are less likely than their non-Indigenous counterparts to receive a transplant. This difference is not fully explained by measurable clinical differences. Previous work suggests that Indigenous Australian patients may be regarded by treating specialists as 'non-compliers', which may negatively impact on referral for a transplant. However, this decision-making is not well understood. The objectives of this study were to investigate: whether Indigenous patients are commonly characterised as 'non-compliers'; how estimations of patient compliance factor into Australian nephrologists' decision-making about transplant referral; and whether this may pose a particular barrier for Indigenous patients accessing transplants.

**Methods:**

Nineteen nephrologists, from eight renal units treating the majority of Indigenous Australian renal patients, were interviewed in 2005-06 as part of a larger study. Thematic analysis was undertaken to investigate how compliance factors in specialists' decision-making, and its implications for Indigenous patients' likelihood of obtaining transplants.

**Results:**

Specialists commonly identified Indigenous patients as both non-compliers and high-risk transplant candidates. Definition and assessment of 'compliance' was neither formal nor systematic. There was uncertainty about the value of compliance status in predicting post-transplant outcomes and the issue of organ scarcity permeated participants' responses. Overall, there was marked variation in how specialists weighed perceptions of compliance and risk in their decision-making.

**Conclusion:**

Reliance on notions of patient 'compliance' in decision-making for transplant referral is likely to result in continuing disadvantage for Indigenous Australian ESKD patients. In the absence of robust evidence on predictors of post-transplant outcomes, referral decision-making processes require attention and debate.

## Introduction

End-stage kidney disease (ESKD) affects Indigenous Australians disproportionately [1]. Transplantation is the optimal treatment [2], but there is a substantial and persistent disparity in transplants given to Indigenous and non-Indigenous patients [3]. The vast majority of Indigenous Australians with ESKD remain on life-long dialysis. Their lower probability of receiving a transplant is not fully explained by measurable clinical differences.

Many Indigenous Australians share contextual factors that may detrimentally affect their engagement with the health system and their response to the significant personal demands imposed by dialysis regimens. As a group, they have higher levels of socioeconomic disadvantage, lower educational attainment and poorer health literacy [4]. Many Indigenous dialysis patients experience isolation and reduced engagement in treatment management [5,6], particularly for those needing to move permanently from rural and remote home communities to obtain dialysis services [7].

With donor kidneys very scarce, clinical decision-making and allocation are often difficult to separate. Practitioners struggle with dual loyalties - to their patient and, to managing a scarce resource [8]. Despite most transplant programs using guidelines to identify suitable candidates [9], decision-making processes are poorly understood [10]. While referring kidney specialists need confidence that the recipient will benefit, the organ shortage might deter them from referring relatively 'risky' patients, irrespective of the benefit those patients may derive.

Referral to a transplant program is a crucial step towards securing a transplant. The nephrologist must identify the patient as potentially suitable and refer him or her for screening. While this sounds straightforward, the decision is within an environment of scarce resource availability, time constraints, imperfect information and contested ethical frameworks for allocation. Geographic remoteness and language and cultural differences further complicate referral. It is necessary to understand referral decision-making within these limitations to assess the barriers facing Indigenous patients.

### 'Compliance' as a predictor of post-transplantation risk

The use of compliance as a predictor of risk may particularly disadvantage Indigenous patients. Previous research suggests that a nephrologist's perception of a patient's 'compliance' with his or her dialysis regimen strongly influences referral. An Australian survey using hypothetical patient vignettes revealed that 'compliance' (not defined in the vignettes) was a highly significant independent predictor of referral - second only to advanced age [11]. The importance of perceived compliance was highlighted in a review of Australian selection guidelines; 'non-compliance' - though poorly defined - is a contraindication, despite a lack of cited evidence, in seven of eight guidelines examined [9]. These studies suggest that nephrologists accord high priority to compliance and commonly see it as a prerequisite for referral.

Perceived non-compliance with pre-transplant dialysis may be seen as a predictor of post-transplant non-compliance. However, the treatment regimens differ markedly. Haemodialysis demands thrice-weekly attendance, strict fluid and dietary control and multiple medications. The post-transplant regimen, by contrast, while requiring strict adherence to medications, has fewer fluid and dietary restrictions and, apart from the immediate post-transplant period, few attendances. While non-compliance with immunosuppressive medications is a recognised cause of transplant failure, any association between pre- and post-transplant non-compliance remains unclear [12-14].

The concept of compliance has been criticised in both the social and medical literature [15,16] in particular because it narrows the perspective on a complex situation [17,18]. Compliance focuses on particular patient behaviours, while often overlooking the influences of systemic and social factors. Behaviours interpreted as poor compliance might stem from factors associated with low socio-economic status and poorly-resourced medical services [19]. The specific social and economic circumstances of many Indigenous Australians might make it difficult for them to maintain the demanding dialysis regimen. Uncertainty about transplant outcomes in this population, together with the chronic organ scarcity, might mean that Indigenous patients are regarded, in general, as too high-risk.

### Exploring nephrologists' views about compliance and suitability

We explored the views of Australian nephrologists about compliance and transplant suitability - both generally and specifically relating to Indigenous patients. This paper draws on in-depth interviews conducted as part of a larger program investigating the barriers facing Indigenous ESKD patients in obtaining transplants. Thematic analysis was undertaken to investigate how and why compliance is factored into decision-making, and the implications for Indigenous patients' obtaining transplants.

## Methods

A large interview study was conducted in 2005-6 as part of the IMPAKT (Improving Access to Kidney Transplants) study [20]. It investigated the reasons why so few Indigenous Australians received transplants, through in-depth interviews of Indigenous and non-Indigenous patients, nephrologists and decision-making staff,. IMPAKT established partnerships with a network of transplant units and dialysis treatment centres treating the vast majority of Indigenous patients. With the support of this network, the IMPAKT team interviewed people in 26 treatment/service centres in metropolitan, regional and remote Australia. A detailed account of the overall study's aims, methodology, ethical issues, recruitment, sampling and data analysis are available elsewhere [20].

The analysis reported in this paper focuses on the interviews with the nephrologists. Two investigators conducted semi-structured, in-depth interviews with 19 nephrologists. In most cases, interviews were conducted individually and in person; three were conducted by telephone. All were audio recorded and later transcribed.

The topics in the nephrologists' interviews included: patient compliance; decision-making about treatment options; assessing suitability for transplant; transplant processes; information and communication processes; and the local renal and health service. This paper focuses on the topic of compliance. The relevant questions explored the views and experiences of nephrologists on compliance and suitability - with a particular focus on Indigenous patients. All questions, with the possible exception of compliance terminology itself, employed value-neutral terms.

Participating nephrologists were asked the following questions about patient compliance:

• Could you comment on what you understand by the term 'compliance' and describe how you would assess whether a patient is compliant?

• Are there any explicit and agreed upon criteria in your unit to determine compliance status? If yes: What are they?

• Are these criteria discussed with patients? At what stage of their treatment would these be discussed?

• How and by whom is non-compliance documented in your unit?

• What - if any - review or management processes operate in your unit for patients who regularly fail to comply with treatment?

• Have you put non-compliant patients on the waiting list and/or recommended them for transplantation? Why *(not)*?

• Are there any particular circumstances or types of patients for whom you make an exception?

• Are there any patient groups (say in terms of age, gender, ethnicity or location) for whom you would say that non-compliance is a particular issue? If yes: Which groups?

• Have you had many non-compliant patients? If yes: What would you say were the causes of their non-compliance?

• Are there particular factors that lead a patient to becoming non-compliant with treatment after transplantation?

• Does this affect your decision about suitability for transplant?

### Participants

Eight large renal units and their associated dialysis service centres, in five State/Territory jurisdictions, were included because they treat the overwhelming majority of Indigenous Australian patients with ESKD. The 19 nephrologists, both general nephrology and transplant physicians, were purposively sampled to include the views of key decision-makers at each site. Relevant nephrologists were invited to attend information sessions at each site and were subsequently invited to participate. There were no Indigenous nephrologists in Australia at the time the IMPAKT study was undertaken so hence all interviewees were non-Indigenous.

### Analysis

The data were analysed through content analysis of the transcribed interviews using QSR NVivo 7 (Doncaster, Australia). The major thematic groups were divided between two investigators who coded all interviews.

Participant demographics were collected via self-reporting. Descriptive statistics were generated using SPSS 15.0 for Windows (Chicago, Illinois).

### Ethical approval

The study was approved by 14 relevant jurisdictional ethics committees, including six all- Indigenous committees. Site-based reference groups operated during the course of the research at each of the nine hospital sites.

## Results

The nephrologists' discussions of compliance and transplant suitability revealed complex decision-making - particularly relating to Indigenous patients. Reliance on compliance was a common, yet contested, issue, which significantly frustrated the nephrologists. The absolute scarcity of organs affected approaches to resource allocation - particularly in relation to the risk of failure. Recurring descriptions relating non-compliance among Indigenous patients, to high-risk status and poor transplant outcomes engendered a sense of caution, and sometimes reluctance, to refer them for transplant.

We explore here five inter-related issues that emerged in the analysis:

• Poor definition and assessment of compliance;

• Uncertainty whether dialysis compliance predicts post-transplant compliance;

• Divergent approaches to the equitable distribution of scarce kidneys;

• Indigenous patients' non-compliance linked to poor outcomes; and

• Conflation of Indigenous non-compliance with "culture".

### Poor definition and assessment of compliance

Asked to explain their understanding of 'patient compliance', most specialists either gave a functional definition of compliance or a description of how they assess compliance. Essentially most referred to a variety of patient behaviours, attitudes and characteristics as markers of compliance, including vaguely defined expectations that patients engage with prescribed treatments and avoid unhealthy lifestyle behaviours. There was no evidence of either a formal definition or a shared understanding of patient compliance among specialists.

Moreover, many were quick to point out flaws in their own methods of assessing compliance which were criticised by several as being subjective, non-quantitative and involving more '*gut-feeling' *[1-4] than measurement. As one explained: *I think it's a subjective assessment . . . which may or may not be accurate*. [5-004]

Nephrologists agreed that compliance was difficult to assess. Although some specialists cited possible assessment methods, most spoke of it in only general terms, if at all. Methods mentioned included clinic attendance records, blood test results, checking medication packs, patient self-reporting, family reporting, prescribing medications known to slow the heart rate and checking the pulse rate, and intuition. However, none were reported as being used consistently within or across units.

### Uncertainty whether dialysis compliance predicts post-transplant compliance

Ambivalence towards the relevance of compliance led to markedly divergent views- both between specialists and within interviews - on the predictive validity of estimations of compliance to identify high-risk candidates.

Several noted that compliance does not translate from dialysis to post-transplant settings because of dissimilarities between the two treatment contexts. The difficulty predicting uptake and maintenance of post-transplant treatments is a source of great concern as explained by one respondent: *[It] is something that really worries us immensely . . . I am totally unable to predict who may be compliant with [post-transplant] medication and who won't be. I've had extraordinary lack of success with predicting that*. [1-11]

Non-compliance was frequently identified as a '*big concern' *[1,2] and a key contraindication to transplantation. Nevertheless, the majority said that they had, indeed, recommended non-compliant patients for transplant. Some nephrologists provided detailed accounts of patients whose 'compliance' status changed after transplantation - in both directions. Several suggested that non-compliant patients pose too much of a risk to receive such a scarce resource. However, even among these specialists there was virtually unanimous agreement that pre-transplant compliance was a *poor *predictor of post-transplant behaviour and outcomes. For example, although saying to one patient: *If you can't comply with dialysis, how do you expect me to trust you to comply with transplantation? I have to give *[transplants] *to patients who are going to take their pills because this is what you have to do*. [1] the nephrologist later went on to say: *I believe that it's very difficult to say if someone's non-compliant on dialysis that they're going to be non-compliant with transplantation. I think the correlation between the two is very difficult and there have been a number of patients whom I've thought over the years would never be compliant, who have been absolutely fine when they're transplanted, so I think that's incredibly difficult to assess*. [1]

Despite obvious frustration, the chief concern of many - particularly in the context of extreme organ scarcity - was forecasting whether or not a patient would maintain the post-transplant regimen. One commented with some resignation: *Attitude to compliance is a major factor and we regard that very closely. Because of the fact that there will never be enough kidneys, if someone doesn't take their medication, that stops someone else, so that's a factor. And I think the hardest thing is how to determine that*. [1]

### Divergent approaches to equitable distribution of scarce resources

The scarcity of donor kidneys was a pervasive theme. Almost every nephrologist identified it as a pressure in decision-making. It clearly shaped many referral practices. Nevertheless there were fundamental differences in nephrologists' approaches to achieving an equitable distribution of kidneys.

Severe organ scarcity creates strong tension between clinician's responsibilities to their individual patient and their perceived (collective) responsibility to manage organ distribution wisely. As one explained: *Not only are we in a position to try and make [patients] better . . . I think we're also paid to safeguard resources, you know, society's resources . . . And although we're trying to make life better for this person, I think we could be judged poorly if we, we squandered something you know . . . I think it's our job and our duty to make sure it's used wisely*. [5-004] Another described the maximising the benefit of the resource, rather than to equity of patient access: *Well I think the key barrier is organ availability and the fact that we're then forced to rank people based on their suitability and potential benefit. Because we, in our unit, have taken up that challenge and not said, "It's too hard", and I think you can say ",It's too hard", and just put everyone on the list. But I think that's probably not what society really needs [us] to do*. [1-10] Another specialist explained that his ultimate responsibility was to the donor: *[Transplant] isn't a right, it's a gift, and the people giving the kidney have a right for it not to be squandered*. [5-004]

Conversely, a handful of specialists did not regard maximizing the utility of donated organs as the chief priority. For them, the benefit to the individual was paramount: *We tend to transplant the Aboriginal patients in the hope that they will be one of the group that does well. Now, you could argue, that that's not very evidence-based, nor is it particularly utilitarian; but I'm not one of those physicians who is bound by evidence, by cost utility . . . I think [Aboriginal patients] deserve to be transplanted, because for those in whom there has been successful transplantation... it's probably a greater advantage for them than it is for the non-Indigenous, because the benefits in terms of cultural and society are greater. They can go back where they came from*. [1-14]

These differences suggest that a '*riskier' *candidate's likelihood of being referred - whether such risk is based on medical, behavioural or circumstantial factors - could depend strongly on where and by whom the patient was being treated.

### Indigenous non-compliance linked to poor transplant outcomes

Indigenous patients were commonly categorised as high-risk candidates. They were also thought to have worse transplant outcomes than non-Indigenous recipients and this belief was frequently associated with the view that such patients had difficulties complying with treatment. As one nephrologist said: '*I think Indigenous patients are over-represented in our non-compliant patients who lose their kidneys from non-compliance*.' [1,2]

The view that Indigenous transplant outcomes were relatively poor impacted on whether and how patients were referred through a heightened sense of caution increased scrutiny or "selectivity" of potential candidates, as reflected here: *I think within the unit, there is a gradual acceptance that a more cautious approach to transplantation has met with better success than the previous [approach] where ... everybody got listed because it was thought unfair to delay Aboriginals. But the outcomes were very unsatisfactory. So I think we are rewarded that a more cautious and selective approach has value*. [1-10]

Concerns about poor outcomes also extended to anxieties about safety in transplanting Indigenous recipients - whether or not transplantation might be considered harmful. As one respondent explained: *Well, that (recent data on post-transplant survival for Indigenous patients) reinforced my pre-existing impression that there is a large number of patients, or a significant proportion of patients from remote areas, who are actually disadvantaged, who actually die by being transplanted*.[5-001]

Poor outcomes were most frequently ascribed to non-compliance. As one respondent stated: *I think the main reason why - even if we think Indigenous patients are suitable - they don't get on the list, is they're at risk of compliance problems*. [1] Asked about outcomes, he said: *I believe they would be worse. I think it's compliance. I think it's nearly always compliance . . . most Indigenous patients' transplants in our unit, and I will say most, ended up with organ loss from (non) compliance. They're a small proportion, so you remember*. [1]

### Conflation of indigenous non-compliance with "culture"

Dialysis non-compliance was often conflated with perceived social and cultural factors. Statements about Indigenous patients being non-compliant and *high-risk *candidates were frequently enmeshed larger, more generalised accounts of their social difficulties and perceived cultural particularities.

Factors repeatedly mentioned included living in a remote location, low socio-economic status, alcohol abuse and low levels of education. The comments of one nephrologist illustrate how situational, cultural and social factors are perceived to contribute: *I think it's partly belief system - whether or not you believe the medication is going to do anything for you or not. Partly it's their tolerability. If medication can cause a consistent adverse effect which you don't like, you're not going to take it. I think it's partly the cost of the medications. If you have to pay, even if you're on a health care card, you have to pay a dispensing fee of $3 or whatever it is. And unfortunately, I think the Indigenous patients are poorer*. [1,2] Another specialist alluded to the challenges of remote living: *I think that compliance is more difficult for people who live remotely. It's just more difficult for them and that doesn't necessarily reflect [on] the individual, it's just it takes a greater level of commitment to be able to comply in that environment*. [1-8]

Cultural issues were also identified as reducing the likelihood of maintaining treatment regimens, including different approaches to time, life priorities, illness, action and responsibility. For example, different approaches to time and to life priorities were thought to factor largely in missed dialysis and appointments. As one nephrologist said: *Aboriginal people don't tend to think too much about tomorrow or next week, they tend to be "now" people. I think therefore it makes it more difficult for them sometimes to understand why they should be taking a tablet for tomorrow*. [1-3] Another, noting the large number of funerals which people attend, said: *so they don't turn up [for dialysis]. Someone's died in their family - they go. Some of these people go to funerals frequently because they've got such a big family - so they don't turn up. And we just accept that it's part of their culture*. [1] The comment inadvertently also highlights the level of illness and loss experienced by Indigenous patients and their families.

The way in which non-compliance and social and cultural circumstances were conflated in the nephrologists' accounts revealed a common pattern of thinking whereby individuals would be pre-judged as high-risk, with accordingly reduced chances of referral. As one nephrologist observed: *I think the perception of illness amongst the Aboriginal people is very, very different, because the perception of time is different and the perception of consequence, of action, is also different - or inaction. I think that really does cloud the whole issue of transplanting the Aboriginal patient and ensuring compliance . . . it would be put forward as a reason not to transplant them by some*. [1-14]

## Discussion

This study explored the views of Australian nephrologists on patient compliance and transplant suitability - both generally and specifically in relation to Indigenous Australians. These interviews afford a unique insight into the complexities of selection and referral of suitable patients and reveal subtle, yet pervasive, barriers to Indigenous patients' receiving kidneys.

There was no evidence that nephrologists relied on a common definition or shared understanding of the notion of patient compliance. They did not use consistent methods of assessing compliance, nor did they consider any methods reliable or particularly useful. Despite it being commonly cited, there was much uncertainty about the predictive validity or value of dialysis compliance to identify risky transplant candidates. While this dilemma frustrated many nephrologists, there was a sense of resignation that a poor predictor of outcome was better than none. Indigenous patients were commonly identified as high-risk and as having relatively poor post-transplant outcomes. Non-compliance with dialysis, often conflated with social and cultural factors, was enmeshed within accounts of Indigenous patients as being 'risky' and was a commonly cited reason for caution in referring them. Without systematically measuring compliance, it is difficult to substantiate or refute claims about non-compliance or its relationship to outcomes.

Recognition of the scarcity of organs permeated the narratives, influencing thinking and referrals. In this context, the nephrologists varied in their approach to decision-making about suitability. Some weighed the perceived risks and benefits for individual patients in isolation, while others considered relative risk compared with other patients. Another recent Australian study, investigating nephrologists' perspectives on waitlisting and allocation of deceased donor kidneys for transplant [21], reported similar tensions. Tong and colleagues found that while nephrologists flagged issues of equity and social justice as important factors in transplant wait-listing and allocation of organs, they felt obliged to refer 'good' patients for transplantation in order to maintain their transplant centre's reputation and furthermore that, in their opinion, the responsibility for ensuring equitable access to transplantation resides with policy makers and the community. In light of these variations in approach, the likelihood of Indigenous patients' - generally considered high risk and non-compliant - being referred could vary significantly depending on where and by whom they were being treated. Furthermore, perceived non-compliance was conflated with generalisations about patients' social and cultural circumstances - a tendency which would likely result in Indigenous patients being considered, almost by default, as high-risk.

The perceived immutability of 'cultural' causes of non-compliance might diminish nephrologists' willingness to recommend Indigenous patients for transplantation. Previous studies of Australian renal care providers and Indigenous ESKD patients suggest that perceptions of non-compliance might stem from miscommunication in cross-cultural encounters, a misalignment in the health paradigms of patients and providers and a lack of appropriate patient education [15,22,23]. While recognising and dealing appropriately with cultural difference is crucial to effective health care provision, the possibility of systemic stigmatisation of patients from minority groups must also be monitored and managed [10,24].

Compounding these concerns are the conflicting views of nephrologists about the relevance of compliance to transplant suitability. Emphasis on the importance of perceived compliance was undermined by the equally widely-held view that pre-transplant compliance is a *poor *predictor of post-transplant behaviour and outcome. With little evidence of such an association [12-14], our findings challenge the validity of factoring compliance so strongly into decision-making and indicate a pressing need for a reconsideration of current selection guidelines and practices.

A further finding was the diversity in nephrologists' views about achieving equitable distribution of kidneys. Strongly opposing views were advocated, based both on *utilitarian *theory - organs should be given to those who would derive the greatest benefit - and *egalitarian *- patients should have equal access and scarce resources should be distributed according to *need*. While it is important that this valuable resource is not wasted, there are serious concerns with a strictly utilitarian approach [24,25]. Ethically (as well as clinically) integrating considerations of risk into patient selection requires wider attention and debate.

This study, drawing on the self-reported views, attitudes and experiences of nephrologists, has limitations. We deliberately explored only the health care provider's perspective, as the 'problem of non-compliance' is arguably located within the culture and views of health care providers, rather than in the views of the patients. The incorporation of the views of a substantial number of senior decision-makers currently managing the majority of Australia's Indigenous ESKD patients enables some firm conjectures to be made about current clinical practice relating to compliance labelling.

## Conclusions

Clinical decision-making relating to patient referral for transplantation is complex. Due to the chronic scarcity of organs, any changes to the criteria will necessarily advantage some patients, while disadvantaging others. Our findings draw attention to a lack of clear evidence regarding predictors of transplant outcomes amongst Indigenous Australians and regarding the use of 'compliance' labelling. Our findings also highlight the need for alternative service delivery models for Indigenous Australian ESKD patients.

A transparent and fair system of patient selection should be a key objective of organ transplantation programs. The development of an agreed national approach to patient selection and organ distribution would assist in realising this goal. A second fundamental step would be the investigation of particular selection criteria to ascertain their relevance and validity in determining patient suitability for transplant. The absence of a strong evidence base to support the inclusion of psychosocial criteria in clinical guidelines highlights the need for research to critically examine the impact of inclusion of particular psychosocial characteristics in assessment of recipient suitability. Is it reasonable to withhold a transplant based upon an assessment of a patient's 'compliance', when there are clear disadvantages for particular patient groups, particularly for Indigenous patients, and when serious concerns exist regarding the legitimacy of using the concept of patient compliance?

Associated with development of patient selection guidelines is the dearth of evidence relating to the most suitable indicators of post-transplant risk and benefit for Indigenous patients. This is a key issue underpinning the continued usage of the problematic concept of compliance in decision-making about kidney transplant suitability. Without a good evidence-base, specialists must rely on inadequate criteria in predicting transplant outcomes for this patient group. In light of the difficulties faced by Indigenous patients in maintaining dialysis treatment, alternative service delivery models that better address the health, social and cultural needs of Indigenous patients should be explored and tested. These service delivery models need to be *regionally specific *to account for the great diversity in settings and contexts across the country. In a wealthy country, with a health system that underpins excellent health outcomes for the majority of its citizens, inequitable access to necessary care and inequitable health outcomes for Indigenous Australians is a fundamentally important and unanswered priority in policy making.

## Competing interests

The authors declare that they have no competing interests.

## Authors' contributions

KA coordinated data management, participated in data collection and data analysis, management and analysis and drafted the manuscript. JD coordinated and participated in data collection and analysis. AC conceived of the study, participated in its design, conducted nephrologists' interviews and participated in data analysis. JC participated in the overall management and design of the IMPAKT program, provided statistical and other technical assistance during data collection and participated in data analysis. CP participated in study design, coordinated the Indigenous community engagement component, participated in data collection, management and analysis. MJ conducted nephrologists' interviews. All authors participated in the drafting and/or critical revision of the manuscript and approved the final version to be published.
